# Conflict adaptation is predicted by the cognitive, but not the affective alexithymia dimension

**DOI:** 10.3389/fpsyg.2014.00768

**Published:** 2014-07-22

**Authors:** Michiel de Galan, Roberta Sellaro, Lorenza S. Colzato, Bernhard Hommel

**Affiliations:** Cognitive Psychology Unit, Leiden Institute for Brain and Cognition, Leiden UniversityLeiden, Netherlands

**Keywords:** Alexithymia, cognitive control, conflict adaptation, Simon effect, Gratton effect

## Abstract

Stimulus-induced response conflict (e.g., in Simon or Stroop tasks) is often reduced after conflict trials—the Gratton effect. It is generally assumed that this effect is due to a strengthening of the representation of the current intention or goal, which in turn increases the degree of stimulus and/or response control. Recent evidence suggests that the motivational signal driving the Gratton effect might be affective in nature. If so, individual differences in either the strength of affective signals and/or the ability to interpret such signals might explain individual differences in cognitive-control adjustments as reflected in the Gratton effect. We tested this hypothesis by relating individual sizes of the Gratton effect in a Simon task to scores on the affective and the cognitive dimension of the Bermond/Vorst Alexithymia Questionnaire (BVAQ)—which we assumed to assess individual differences in affective-signal strength and ability to interpret affective signals, respectively. Results show that the cognitive, but not the affective dimension predicted control adjustment, while the accuracy of heartbeat detection was only (and only weakly) related to online control. This suggests that the motivation to fine-tune one's cognitive-control operations is mediated by, and may depend on one's ability to interpret one's own affective signals.

## Introduction

Traditional views on the role of motivation in action control have focused on the process of decision-making, that is, on the selection of goals that an agent intends to pursue (e.g., Kahneman, 2011) and, in few cases, on the evaluation of performed actions with respect to that goal (e.g., Miller et al., 1960; Achtziger and Gollwitzer, 2008). According to this perspective, motivational processes keep themselves busy with the “what” of actions and provide action-control processes with information about wanted action outcomes. More recent research has suggested a somewhat more complex picture, however—a picture indicating that motivational processes also affect the “how” of action control.

A prime example is the so-called “conflict-adaptation effect” or “Gratton effect” (the theoretically more neutral term, after Gratton et al., 1992), that is observed in tasks where irrelevant stimuli or stimulus features induce response conflict (such as the Simon or Stroop task). Conflict-inducing trials (i.e., trials in which the irrelevant stimulus or stimulus feature primes an incorrect response; i.e., response-incompatible trials) are known to increase reaction times (RTs) and error rates (PEs) as compared to neutral trials or trials in which stimuli prime the correct response (compatible trials). The Gratton effect consists in the observation that the size of such compatibility effects varies as a function of the compatibility in the *previous* trial: the compatibility effect in the present trial is commonly larger after a compatible trial than after an incompatible trial (Gratton et al., 1992; Stürmer et al., 2002). This observation has been taken to reflect the conflict-induced adaptation of cognitive control (Botvinick et al., 2001): control relaxes after a trial without conflict (i.e., after a compatible trial) but is strengthened after a conflict trial (i.e., after an incompatible trial), so that control is “hit” more by conflict after compatible than incompatible trials. Even though there are reasons to assume that the Gratton effect is not a pure measure of adaptivity (Hommel et al., 2004; Schmidt and De Houwer, 2011), the current understanding is that it does reflect control adaptations to some degree (e.g., Verguts and Notebaert, 2009).

From a motivational point of view, the Gratton effect raises two questions. First, why do people need to adapt the degree to which they are controlling their actions at all? If they would keep control at some optimum, adaptation should not be necessary. The fact that they apparently do not suggests that they are either unable to do that, perhaps because cognitive control relies on limited and quickly depleting resources (Baumeister, 2002), or not willing to invest 100% of the available resources (De Jong et al., 1999; Kool and Botvinick, 2014), perhaps because that would be wasting energy in non-conflict trials. Indeed, several observations indicate that people spontaneously reduce the amount of cognitive effort they invest in a task over time and trials (De Jong et al., 1999; Altmann, 2002; Kool and Botvinick, 2014; Kool et al., 2014), which suggests the operation of an energy-saving motivational process realizing a kind of “law of least effort” (Hull, 1943). Even though most of the related research is rather recent, the general idea that cognitive-control resources are flexibly tailored to the task demands is an old one that goes back to Hillgruber's (1912) “difficulty law of motivation” (Brehm and Self, 1989; Hommel et al., 2012).

The second important motivational question is why people increase the amount of cognitive resources they invest after conflict trials. Conventional motivational accounts would suggest that control adaptation is driven by action errors, because these would imply a mismatch between the selective goal and the actual outcome, but not by mere conflict. This would allow for learning through errors but not for the prevention of errors through the anticipatory adjustment of action-control parameters that the Gratton effect is taken to reflect. According to Botvinick et al. (2001), the necessity of control adjustments is signaled by the anterior cingulate cortex (ACC), which receives input representing the current degree of response conflict. In contrast, Holroyd and Coles (2002) have emphasized the role of expected or actual negative feedback in motivating control adjustments. Botvinick (2007) pointed out that these two views might not be incompatible if one assumes that conflict serves as a teaching signal for avoidance-learning processes.

Interestingly, all three approaches imply that affective states might be involved in driving control adjustments: Just like real or anticipated negative events induce negative affect, conflict between response tendencies has been assumed to have the exact same effect (Festinger, 1957). This suggests that control adjustments are driven by negative affect. Consistent with this prediction, van Steenbergen et al. (2009, 2010, 2012a) found that the Gratton effect is reliable after a negative-mood induction or after trials providing unexpected financial loss, but absent after positive-mood induction or unexpected reward. Along the same lines, Holmes and Pizzagalli (2007) showed that participants with high scores in depressive symptoms were associated with impaired post-error and post-conflict performance adjustments. Similarly, van Steenbergen et al. (2012b) found that, in depressive patients, depressive symptoms induced by acute tryptophan depletion were associated with increased conflict adaptation. It is important to emphasize that in none of these studies were the affective states related to actual action outcome, error rates, or objective success. This means that affective states are not just a byproduct of response conflict or the expectation of failure but rather the actual motivational force that is driving action-control adjustments. In other words, control adjustments seem to be mainly driven by (negative) affect—or, more specifically, by the (negative) sum of positive and negative affective signals (van Steenbergen et al., 2009, 2010). If so, response conflict or negative event expectations can be considered just examples of means to induce a negatively-biased affect state (see also Dreisbach and Fischer, 2012).

If control adjustments would really be driven by negative affect, one would expect that interindividual differences in experiencing or processing affect predict differences in control adjustments—and this was indeed the hypothesis we tested in the present study. We considered two ways in which affect processing might differ.

For one, people may differ with respect to the strength of the signal that affect-related systems send to systems sensing the need for control, such as the ACC. Indeed, Critchley et al. (2004) reported that people's accuracy in reporting their own heartbeat was positively correlated with activation of their insular cortex—a structure that integrates affective and motivational signals and “reports” to the ACC (Craig, 2009; Critchley and Harrison, 2013). Likewise, van Veen et al. (2009) found that the degree of attitude change in a cognitive-dissonance task was positively correlated with activation in the insular cortex. Hence, it might be that some individuals generate stronger affective signals than others, so that their action-monitoring systems would be more likely to pick up signals indicating negative affect. As a consequence, these individuals would be expected to show a more pronounced Gratton effect.

For another, people may differ with respect to the degree that they are able to interpret signals sent by affect-related systems. Indeed, several authors have argued that affectively relevant signals might often be difficult to interpret and people may differ with respect to their ability to attribute them to the actual cause. For instance, Schachter (1971) provided evidence that eating behavior in obese individuals is triggered more by external, environmental cues than in normal-weight people, who rely more on internal, hunger-related signals—presumably because the former find it difficult to tell hunger-related from hunger-unrelated signals. Along the same lines, Duncan and Laird (1997) reported systematic differences between participants with respect to the degree to which their mood was affected by facial-expression manipulations: while some people felt happier when activating affect-related facial muscles involved in smiling and more angry when activating muscles involved in frowning, others were unaffected by any manipulation. As the authors suggest, this might reflect differences in the ability to interpret proprioceptive cues. Applied to action control, these considerations suggest that people could differ with respect to their ability to interpret internal affective signals. If so, people who are more able to interpret such signals should be more likely to identify negative affective states and, thus, show a more pronounced Gratton effect.

In the present study, we used the Bermond/Vorst Alexithymia Questionnaire (BVAQ; Vorst and Bermond, 2001) to assess individual differences with respect to both the strength of affective signals and the ability to interpret them. The BVAQ has two major dimensions: an affective and a cognitive dimension. While the affective dimension can be taken to reflect the perceived intensity and frequency of affective signals (Wiens et al., 2000; Herbert et al., 2007), the cognitive dimension assesses the degree to which people engage in analyzing, identifying, and communicating their affective states (Silani et al., 2008; Bermond et al., 2010). Accordingly, we hypothesized that a reliable correlation between the affective BVAQ score and the individual size of the Gratton effect would indicate a role of individual differences in affective signal strength. In contrast (or in addition), a correlation between the cognitive BVAQ score and the Gratton effect would suggest a role of individual differences in the ability to interpret affective signals. Another measure we considered was the individual accuracy in a heartbeat-detection task (e.g., Pollatos and Schandry, 2004). As mentioned already, accuracy in such tasks has been shown to correlate with the degree of insular-cortex activity (Critchley et al., 2004), suggesting that it might represent an alternative measure of signal strength.

To investigate the adaptivity of cognitive control, we used a standard conflict-inducing task, the Simon task (for reviews, see Lu and Proctor, 1995; Hommel, 2011). The Simon effect is observed when people respond with spatially defined responses to non-spatially defined features. If the location of the stimulus, which is nominally irrelevant to the task, happens to correspond to the location of the correct response, responses are quicker and more accurate than if the locations of stimulus and response do not match. The effect is commonly attributed to response conflict created by the automatic, stimulus-induced activation of the incorrect response in stimulus-response-incompatible trials. The Simon task has been shown to give rise to reliable Gratton effects (Stürmer et al., 2002), which made it suitable for our purposes.

## Methods

### Participants

Sixty students (8 males; mean age 19.7 years) of the Leiden University participated in the experiment for partial fulfillment of course credit (6.50 Euro). Written informed consent was obtained from all subjects; and the protocol was approved by the local ethical committee (Leiden University, Institute for Psychological Research).

### Procedure

Participants estimated their heartbeat and completed the questionnaire in a counterbalance order. Afterwards, they performed the Simon task.

#### Heartbeat detection

Heartbeat detection was measured using the Mental Tracking Method (e.g., Tsakiris et al., 2011), which has been widely-used to assess interoceptive sensitivity, correlates highly with other heartbeat detection tasks, and has good test-retest reliability (Knoll and Hodapp, 1992). Participants were asked to remove any nail polish and clean their finger with an alcohol swab. Heartbeat assessment was measured with a finger type pulse oximeter (Contec CMS50D+; accuracy = 2 bpm/2%). The display on the device was taped off, making it unreadable to the participant. The device was connected to the thumb of the participant's non-dominant hand and attached with a USB cable to a PC running Windows™. Computer software was used to display and record (average) heartbeat rate, not visible to the participant. Participants were seated in a chair, in a still and relaxed position without speaking, and instructed to report their pulse rate as detected by interoception. Touching their wrist or other body parts that would reveal their pulse was not allowed. Before measurement participants were seated without speaking for 2 min, then a 15-s practice trial was performed. The task consisted of four trials of different duration (25, 35, 45, and 100 s), the sequence of which was randomized, balanced, and unknown to the participant beforehand. The experimenter indicated the beginning and end of the trial by an aural (START/STOP) and visual (hand) signal. After each trial participants were asked to verbally report what they thought that their average heartbeat had been. No feedback was given on performance. There was a 30 s rest interval between each trial.

#### BVAQ

A paper version of the Dutch BVAQ was used (Vorst and Bermond, 2001). Participants replied to emotion-related statements on 5-point Likert scales. The questionnaire consists of 40 questions assessing alexithymic tendencies on two dimensions: cognitive and affective.

The cognitive dimension comprises of three subscales: verbalizing emotions, identifying emotions, and analyzing emotions. The “verbalizing emotions” subscale concerns the degree to which one is able or inclined to describe or communicate about one's emotional reactions (e.g., “I find it difficult to verbally express my feelings”). The “identifying emotions” subscale concerns the degree to which one is able to define one's arousal states (e.g., “When I am distressed, I know whether I am afraid or sad or angry”). The “analyzing emotions” subscale concerns the degree to which one seeks out explanations of one's own emotional reactions (e.g., “I hardly ever go into my emotions”). Subscales have 8 questions each, scored from 1–5. Possible scores thus range from 24–120 (8–42, clearly non-alexithymic; 43–61, modal; and 62–120, alexithymic).

The affective dimension comprises of two subscales: emotionalizing and fantasizing. The “emotionalizing subscale” concerns the degree to which someone is emotionally aroused by emotion-inducing events (e.g., “When something totally unexpected happens, I remain calm and unmoved”). The “fantasizing” subscale concerns the degree to which someone is inclined to fantasize, imagine, daydream, etc. (e.g., “Before I fall asleep, I make up all kinds of events, encounters, and conversations”). Subscales have eight questions each, scored from 1–5. Possible scores therefore range from 16–80 (16–28, clearly non-alexithymic; 29–41, modal; 42–80, alexithymic).

#### Simon task

The Simon task was performed on a computer running Windows™, attached to a 17″ color monitor. Viewing distance was about 60 cm. A continuously centrally-displayed small (0.5 cm) dark gray square served as fixation point. Stimuli were green and blue circles (1.5 cm in diameter), presented to the left or right of fixation. The color and location of the stimuli varied randomly and equiprobably. Circles stayed on the screen until 1500 ms had passed or a response was given. Intervals between stimuli varied randomly between 1250–1750 ms, in steps of 100 ms. Feedback on PEs and RTs was provided at the end of each block. Responses were made by pressing the “z” or “?” buttons of the QWERTY computer keyboard with the left or right index finger, respectively. Participants were instructed to react as fast and as accurate as possible to the stimulus-color, but not location.

#### Data analysis

As a manipulation check (to demonstrate reliable Simon and Gratton effects), mean RTs and PEs from the Simon task were submitted to separate repeated-measures ANOVAs, with compatibility in present trial (compatible vs. incompatible) and compatibility in previous trial (compatible vs. incompatible) as factors.

For the correlation analyses, seven scores were calculated: (1) the size of the Simon effect in RT (RT incompatible in the present trial minus RT compatible in the present trial); (2) the size of the Simon effect in PE (incompatible minus compatible); (3) the size of the Gratton effect in RT (Ci[incompatible trial following compatible trial] – Cc[compatible trial following compatible trial]) – (Ii[incompatible trial following incompatible trial] – Ic[compatible trial following incompatible trial]); (4) the size of the Gratton effect in PE ((Ci – Cc) – (Ii– Ic)); (5) the BVAQ score for the affective dimension (emotionalizing subscale + fantasizing subscale, a higher score indicating more alexithymia); (6) the BVAQ score for the cognitive dimension (verbalizing subscale + identifying emotions subscale + analyzing subscale, a higher score indicating more alexithymia); and (7) an interoceptive awareness score, calculated from the four different heartbeat detection intervals [¼ Σ(1 – (|recorded heartbeats–counted heartbeats|)/recorded heartbeats)]; this score can vary between 0 and 1, with higher scores indicating better heartbeat detection.

## Results

### Heartbeat detection and BVAQ

Judgment accuracy in heartbeat detection varied between 0.46 and 0.98 (Mean = 0.82, *SD* = 0.14).

The BVAQ scores varied between 25 and 78 (Mean = 51, *SD* = 13) for the cognitive dimension, and between 18 and 60 (Mean = 36, *SD* = 9) for the affective dimension.

### Simon task (manipulation check)

As the Simon effect is sensitive to the overall RT level (Hommel, 1993), participants with mean RTs exceeding 2.5 *SD* were excluded; this led to the exclusion of two participants [females; Mean_(Cognitive Dimension)_ = 50, *SD* = 4, Mean_(Affective Dimension)_ = 46, *SD* = 9]. As usually found, performance on the Simon task was faster (375 ms vs. 411 ms), *F*_(1, 57)_ = 126.41, *p* < 0.001, η^2^_p_ = 0.69, and more accurate (2.7 vs. 6.6%), *F*_(1, 57)_ = 47.96, *p* < 0.001, η^2^_p_ = 0.46, on compatible than on incompatible trials. A standard Gratton effect was obtained, as indicated by a significant interaction between previous trial and present trial compatibility both for RTs, *F*_(1, 57)_ = 190.65, *p* < 0.001, η^2^_p_ = 0.77, and PEs, *F*_(1, 57)_ = 93.36, *p* < 0.001, η^2^_p_ = 0.62. The Simon effect in RTs was significant after compatible trials (65 ms), *F*_(1, 57)_ = 328.21, *p* < 0.001, η^2^_p_ = 0.85, but not after incompatible trials (6 ms), *F* = 2.49, *p* = 0.12. For the PE data, a regular Simon effect (1.1 and 10.1% in compatible and incompatible trials, respectively) was observed after compatible trials, *F*_(1, 57)_ = 110.41, *p* < 0.001, η^2^_p_ = 0.66, while a reverse, but not significant, effect was observed after incompatible trials (4.3 and 3.0% in compatible and incompatible trials, respectively), *F*_(1, 57)_ = 3.71, *p* = 0.06, η^2^_p_ = 0.06.

### Correlation analyses

As the correlation analyses had the goal to determine whether alexithymia measures can predict *existing* Simon and Gratton effects (rather than explaining their absence/inversion, an issue beyond the scope of the present study), participants with negative Simon or Gratton effects were excluded from analysis; this led to the exclusion of three participants [2 females with a negative Simon effect and 1 male with a negative Gratton effect; Mean_(Cognitive Dimension)_ = 56, *SD* = 19, Mean_(Affective Dimension)_ = 44, *SD* = 12].

The BVAQ scores for the 55 participants included in the correlation analyses varied between 25 and 78 (Mean = 50, *SD* = 13) for the cognitive dimension, and between 18 and 60 (Mean = 35, *SD* = 9) for the affective dimension.

**Figure 1:**
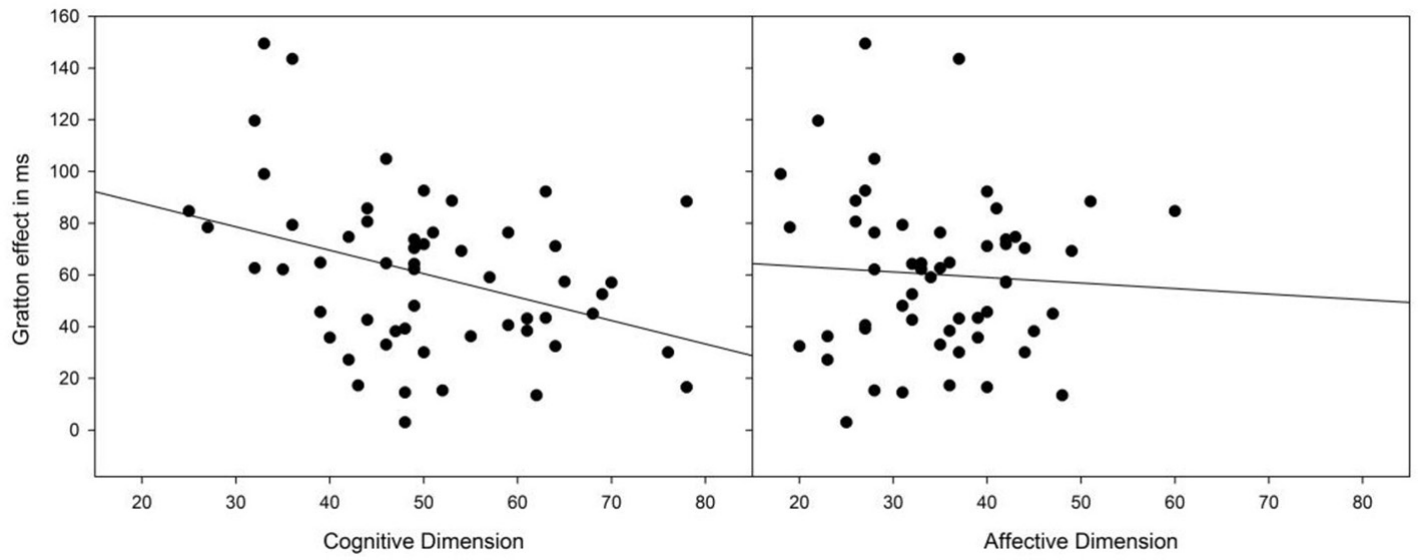
**Scatterplots of the Gratton effect against BVAQ scores for the cognitive and affective dimensions**. The BVAQ cognitive, but not the affective dimension predicts the degree to which people adapt their cognitive control to task demands, as measured by the Gratton effect.

Table 1 provides an overview of the correlations between the two BVAQ dimensions (and their respective subscales) and our critical measurements (i.e., heartbeat-detection score and the Simon and the Gratton effects in terms of RTs and PEs). Notably, the Gratton effect in RT was reliably predicted by the cognitive (*r* = −0.37, *p* = 0.005), but not by the affective dimension (*r* = −0.061, *p* = 0.66) of the BVAQ (see Figure 1). The significant correlation between the Gratton effect and the cognitive dimension of the BVAQ was mainly due to two of the three cognitive subscales: verbalizing (*r* = −0.37, *p* < 0.01), and analyzing (*r* = −0.28, *p* < 0.01). That is, the less people experience problems in verbalizing and analyzing their emotions the more likely they engage in adapting the degree of cognitive control to task demands. To evaluate the robustness of these results, we applied Bayesian statistic to the Pearson's correlation coefficients computed between the Gratton effect in RT and the two BVAQ dimensions, to provide direct support for either the null (H_0_) or the alternative (H_1_) hypothesis. To this end, we used the Bayesian correlation method proposed by Wetzels and Wagenmakers (2012; see also Liang et al., 2008) to compute the Bayes factor (BF; Jeffreys, 1961)—an index that quantifies the relative plausibility of the data under the two competing hypotheses. A BF greater than 1 represents evidence for H_1_, whereas a BF smaller than 1 represents evidence for H_0_ (see Jeffreys, 1961, for a coarse classification). Bayesian analyses confirmed our results. For the cognitive dimension, the analysis yielded a BF of 5.0—substantial evidence for H_1_ (cf. Jeffreys, 1961). In contrast, for the affective dimension, the analysis yielded a BF of 0.1–substantial evidence for H_0_.

**Table 1:** **Correlations between the two BVAQ dimensions (and their respective subscales) and the heartbeat-detection score, the Simon and Gratton effects (in terms of RTs and errors)**.

	**Simon effect**	**Simon effect error**	**Gratton effect**	**Gratton effect error**	**Heartbeat perception**
Cognitive dimension	0.207	−0.112	−0.370^**^	−0.205	−0.081
Verbalizing	0.267^*^	−0.117	−0.366^**^	−0.238	−0.169
Identifying emotions	0.128	−0.046	−0.186	−0.132	0.096
Analyzing	0.012	−0.091	−0.282^*^	−0.050	−0.114
Affective dimension	0.048	−0.022	−0.061	−0.190	−0.051
Emotionalizing	0.128	−0.129	−0.078	−0.124	−0.175
Fantasizing	−0.021	0.054	−0.026	−0.156	0.048

* p < 0.05;

** *p < 0.01*.

Interestingly, we observed only little evidence of a relationship between the Simon effect and alexithymia scores: apart from a just-significant positive correlation between the size of the Simon effect in RTs and the verbalizing subscale of alexithymia (*r* = 0.27, *p* = 0.049), none of the alexithymia subscales (nor the cognitive and affective dimensions) correlated with this measure (*p*_s_ ≥ 0.13; see Table 1).

Also of interest, the heartbeat-detection score did not correlate with any of the alexithymia dimensions (or any subscale, *p*_s_ ≥ 0.20; see Table 1). Furthermore, this measure did not correlate either with the Simon effects in RTs and PEs or the Gratton effect in RTs (*p*_s_ ≥ 0.28). However, a significant positive correlation between heartbeat detection and the Gratton effect in PEs (*r* = 0.27, *p* = 0.043) suggested that more accurate heartbeat detectors are more likely to engage in cognitive control adjustments aimed at preventing errors in conflict-inducing trials.

Finally, we obtained positive correlations between the Simon effects in RTs and PEs (*r* = 0.35, *p* < 0.01), and between the Gratton effects in RTs and PEs (*r* = 0.31, *p* < 0.05).

## Discussion

The aim of our study was to better characterize the motivational signal driving cognitive-control processes to adapt to task demands. Given the evidence that this signal is related to negative affect, we reasoned that people may differ with respect to either the strength of the signal provided by affect-related neural systems (such as the insular cortex; Critchley et al., 2004) or the individual ability to interpret signals of that sort (e.g., Schachter, 1971). We assessed the adaptivity of cognitive control by means of the Gratton effect in a Simon task and considered the affective and cognitive dimensions of the BVAQ to reflect individual differences in signal strength and interpretational abilities, respectively.

The outcome is clear in showing that individual differences in the Gratton effect are associated with the cognitive, but not the affective BVAQ dimension. This suggests that the ability or preference to analyze and properly interpret affect signals is necessary for engaging in adaptive control. It is important to point out that the BVAQ assesses the subjective experience of difficulties in analyzing and interpreting one's own emotions but does not indicate whether the individual interpretations are correct. Hence, our findings do not rule out the possibility that people engage in the trial-to-trial fine-tuning of cognitive control only because they think they are good in interpreting their feelings while they actually are not. If so, interpretational optimism might be particularly motivating for engaging in the optimization of control, while the actual availability of interpretational skills might not matter so much. And yet, given that life provides numerous occasions to provide objective feedback about the validity of one's interpretation of one's own emotions (Wittgenstein, 1977), it makes sense to assume that the subjective responses to the BVAQ do bear some relationship to the objective abilities. If so, this would suggest that some degree of skill in interpreting one's own affective signals is necessary to exert efficient cognitive control or at least diagnostic for the ability to do so.

One possibility is that the cognitive, perhaps even conscious, interpretation of affective signals is a necessary processing step intervening between the generation or emergence of conflict and operations aiming at reducing or eliminating such conflict in the future. Individuals who can interpret such signals more efficiently, for instance by being better able to discriminate between positive and negative signals, would thus be more effective in adapting cognitive-control settings—as assessed by the Gratton effect. Note that this would not necessarily mean that they can avoid conflict in the first place, even though the positive correlation between verbalizing and the Simon effect in PEs suggests that they might to some degree.

It might be objected that, even though many studies in this area suffer from problematic flaws that make the interpretation of their outcomes difficult (Desender and Van den Bussche, 2012), there is some evidence that conflict adaptation can occur in the absence of conscious experience of the conflict-inducing stimuli (Desender et al., 2013; Reuss et al., 2014). However, note that the lack of consciousness regarding the source of conflict-induced affect does not necessarily imply the absence of conscious experience of the resulting affective state: people do consciously experience affect even if that was induced by an unconscious event (Winkielman et al., 2007). Hence, it might well be that cognitive, perhaps even conscious interpretation of affective signals, is necessary to drive conflict adaptation. However, it would be equally possible that conscious interpretation and (unconscious?) conflict adaptation merely rely on the same information. For instance, subcortical mechanisms might send affective signals that vary in discriminability and diagnostic validity. Low discriminability/diagnosticity would impair both conscious judgments about one's own affective state and the possibly unconscious, automatic integration of affective signals for conflict adaptation. Accordingly, measures of the former (as the cognitive alexithymia scale) would correlate with assessments of the latter (as the Gratton effect), even if affective judgment and conflict adaptation would not causally impact each other. But even in that case, the ability to consciously interpret one's own affective states would still be diagnostic of one's conflict-adaptation abilities.

It is interesting to note that the heartbeat-detection score did not correlate with any of the alexithymia scores. Previous findings have shown that heartbeat detection accuracy is correlated with the same measures as at least some alexithymia subscales (e.g., activity in the insular cortex; see Critchley et al., 2004; Silani et al., 2008; Herbert et al., 2011), which is why we considered a direct correlation between heartbeat detection scores and alexithymia at least possible. The fact that we did not observe such a correlation might suggest that the heartbeat-detection task reflects different aspects of affective signal production than the affective dimension of the BVAQ. Moreover, Wiens et al. (2000) found that visceral perception plays a role in the experience of the intensity of emotions. Given that the intensity of emotions is related to the affective dimension of alexithymia, this may explain why we found a correlation between the Gratton effect and the cognitive scale of alexithymia, but not between the Gratton RT effect and heartbeat detection (note that we did find a correlation for Gratton PE effect). However, there are reasons not to over-interpret the absence of a reliable correlation in a relatively small sample, so that we are reluctant to draw strong conclusions. For instance, the mean heartbeat detection accuracy in our study was 20% points higher than in previous studies (see Critchley et al., 2004; Tsakiris et al., 2011), suggesting that our participants might have been too good (i.e., produced a ceiling effect) to allow for a reliable correlation.

In any case, our findings suggest that the individual motivation to engage in the fine-tuning of cognitive-control processes is related to the perceived and/or objective ability to interpret one's own affective states. This provides further evidence for an important role of emotion in the control of cognition and action, and a deeper insight into the way cognitive control operations are motivated. However, it is important to note that our results can be generalized only to a population showing regular (i.e., positive) Simon and Gratton effects, and cannot be extended to the individuals exhibiting uncommon negative effects.

### Conflict of interest statement

The authors declare that the research was conducted in the absence of any commercial or financial relationships that could be construed as a potential conflict of interest.
