# Supplementary material for: Conflict adaptation is predicted by the cognitive, but not the affective alexithymia dimension
Source: Front Psychol. 2014 Jul 22;5:768. doi: 10.3389/fpsyg.2014.00768 (PMC4106422; doi:10.3389/fpsyg.2014.00768)
Supplement: Supplementary file 1 [file DataSheet1.ZIP › Alexithymia & Conflict adaptation/Important information.docx]

**Conflict adaptation is predicted by the cognitive, but not the affective alexithymia dimension**

Michiel de Galan^‡^

Roberta Sellaro^‡^

Lorenza S. Colzato

Bernhard Hommel*

(^‡^shared first author)

Leiden University

Cognitive Psychology Unit & Leiden Institute for Brain and Cognition

Leiden, The Netherlands

**Abstract**

Stimulus-induced response conflict (e.g., in Simon or Stroop tasks) is often reduced after conflict trials—the Gratton effect. It is generally assumed that this effect is due to a strengthening of the representation of the current intention or goal, which in turn increases the degree of stimulus and/or response control. Recent evidence suggests that the motivational signal driving the Gratton effect might be affective in nature. If so, individual differences in either the strength of affective signals and/or the ability to interpret such signals might explain individual differences in cognitive-control adjustments as reflected in the Gratton effect. We tested this hypothesis by relating individual sizes of the Gratton effect in a Simon task to scores on the affective and the cognitive dimension of the Bermond/Vorst Alexithymia Questionnaire (BVAQ)—which we assumed to assess individual differences in affective-signal strength and ability to interpret affective signals, respectively. Results show that the cognitive, but not the affective dimension predicted control adjustment, while the accuracy of heartbeat detection was only (and only weakly) related to online control. This suggests that the motivation to fine-tune one’s cognitive-control operations is mediated by, and may depend on one’s ability to interpret one’s own affective signals.

**Important Information**

Folders:

- Simon Raw: Raw data in the Simon task [The data were collected in two identical cabins (Lab: SB08 in FSW building, Pieter de la Court gebouw, Leiden; cabin 2 and cabin 4. Note that, due to a mistake in assigning numbers to participants, participant #11 in cabin 2 is actually the # 12]. Important: This folder contains the manual of the task (folder Simon_Task) used to run the experiment, which is necessary to analyze the data.
- Simon Task: Simon task experiment used in this study to generate trials and collect data
- SPO2 HBP objective measures: output of the Mental Tracking Method used to measure heart beat
- HBP subjective Measure: Participants’ subjective estimation of their heart beat. The file also contains the interoceptive awareness score for each participant, calculated from the four different heartbeat detection intervals [¼ Σ(1 – (|recorded heartbeats–counted heartbeats|)/recorded heartbeats)]. Note that participant # 61 completed the alexithymia questionnaire and the heart beat detection test but not the Simon task. # 42, 50 and 62 correspond to non-showed up participants.
- Alexithymia & Gratton_All data: Spss file containing all data (i.e., measures derived from the Simon task, alexithymia scores, interoceptive awareness scores, age, and sex). Please, pay attention to the labels associated with each variable.
